# FBXO17 Inhibits the Wnt/β-Catenin Pathway and Proliferation of Ishikawa Cells

**DOI:** 10.7150/ijms.60335

**Published:** 2022-08-15

**Authors:** Zi-Meng Zheng, Ying-Ying Wang, Min Chen, Hui-Li Yang, Zhen-Zhen Lai, Ming-Qing Li, Jun Shao

**Affiliations:** 1 Insitute of Obstetrics and Gynecology, Hospital of Obstetrics and Gynecology, Fudan University, Shanghai 200082, People's Republic of China.; 2 NHC Key Lab of Reproduction Regulation (Shanghai Institute of Planned Parenthood Research), Hospital of Obstetrics and Gynecology, Fudan University, Shanghai 200082, People's Republic of China.; 3 Department of Obstetrics and Gynecology, Yidu Central Hospital of Weifang, Weifang 262500, People's Republic of China.; 4 Shanghai Key Laboratory of Female Reproductive Endocrine Related Diseases, Hospital of Obstetrics and Gynecology, Fudan University, Shanghai 200011, People's Republic of China.

**Keywords:** FBXO17, endometrial cancer, Wnt/β-catenin pathway, malignant progression, Ishikawa

## Abstract

Uterine corpus endometrial carcinoma (UCEC) is one of the most common types of cancer in women, and the incidence is rapidly increasing. Studies have shown that various signaling pathways serve crucial roles in the tumorigenesis of UCEC, amongst which the Wnt/β-catenin pathway is of great interest due to its crucial role in cell proliferation and the huge potential as a therapeutic target. In the present study, it was shown that FBXO17, which is a member of the F-box family, is abnormally downregulated in UCEC tissues compared with non-tumor endometrial tissues, and this was significantly associated with the clinical histological grade, as well as the abnormal proliferation of the UCEC cell line, Ishikawa, both* in vitro* and* in vivo*. Besides, the results suggested that FBXO17 may inhibit the Wnt/β-catenin signaling pathway and influence the expression of adhesion molecules, such as E-cadherin and N-cadherin in Ishikawa cells. In conclusion, these findings indicate that FBXO17 is a novel inhibitor of endometrial tumor development and it likely exerts effects via regulation of the Wnt signaling pathway.

## Introduction

Due to an increasingly ageing population and an increase in the proportion of obese individuals, the incidence of uterine corpus endometrial carcinoma (UCEC) is rising 1. It is estimated that by 2030, the morbidity of UCEC in the USA will be 55% higher than it was in 2010 2.

Treatment selection of UCEC is primarily based on histological classification and the pathological stage; however, identification of specific molecular targets may pave the way for more individualized treatment regimens 3, 4. The Wnt/β-catenin pathway is dysregulated in several types of cancer, and has received significant attention in studies on tumorigenesis 5. When the canonical Wnt signaling is not activated, β-catenin is phosphorylated by a protein complex composed of glycogen synthase kinase-3 (GSK-3), and this phosphorylation ultimately results in the degradation of β-catenin 6. The above process is inhibited as a result of binding of Wnt to the Frizzled receptor on the cell membrane 7, after which, β-catenin accumulates and promotes the transcription of direct target genes, such as *Cyclin D1* and *C-MYC* 8. Activation of the Wnt/β-catenin pathway serves a vital role in the normal developmental process, but the dysregulation of this pathway is associated with tumorigenesis 9. Previous studies have reported that the gene encoding β-catenin is commonly mutated in low-stage endometrioid UCEC patients with increased risk of recurrence 1, 10, 11, and nuclear accumulation of β-catenin appeared in 12-31% UCEC patients 12. Several Wnt signaling inhibitors were investigated in UCEC but only few progressed to clinical trials 12-15.

The ubiquitin-proteasome system is one of the most important methods of protein degradation in eukaryotes, and is essential in maintaining homeostasis of the intracellular protein levels 16. This system consists of 4 parts, of which the E3 ubiquitin ligase is the best studied 17. The F-box protein acts as a substrate recognition subunit in the SKP1-Cullin1-F-box (SCF), one of E3 ubiquitin ligase complexes, and >70 different F-box proteins have been verified to regulate various biological processes, such as cell-cycle control 18, 19. Also, it has been widely reported that the dysregulation of F-box proteins causes tumorigenesis 20, 21.

FBXO17 is an F-box protein that was first identified in 2002 22, but its function has only been determined in several diseases more recently 23-27. However, there is still lack of studies investigating this protein and its function in UCEC. In the present study, it was shown that the expression of FBXO17 was abnormally decreased in patients with UCEC. Thus, we chose one of UCEC cell line Ishikawa to investigate its effect on the proliferation and its possible target pathways.

## Materials and methods

### The cancer genome atlas (TCGA) data analysis

RNA-Seq expression data of UCEC group (n=552) or healthy control group (n=35) were downloaded from the TCGA database (https://cancergenome.nih.gov/). Overall survival curve of UCEC patients with high/low level of FBXO17 was drawn using the GEPIA2 database (http://gepia2.cancer-pku.cn/) based on TCGA database via Kaplan-Meier analysis.

### Patient samples

A total of 40 UCEC tissues and 15 non-tumor endometrium tissues from Fudan cohort were used to assess the FBXO17 mRNA expression levels. The clinicopathological characteristics of the recruited patients from whom tissues were obtained are shown in Table 1. Additionally, 90 endometrial carcinoma and paracarcinoma tissues were used for immunohistochemistry analysis and were obtained from the tissue bank of Obstetrics & Gynecology Hospital of Fudan University from patients who underwent surgery between 2015 and 2018. Amongst those tissues, UCEC tissues were obtained from patients who were diagnosed with FIGO I-IV stage UCEC, but no other primary tumors and normal tissues were extracted from non-tumor patients who underwent a hysterectomy. All tissues were stored in liquid nitrogen after removal for further experiments. Clinical information was also collected from the above 90 endometrial carcinoma patients.

The present study was approved by the Ethics Committee of Obstetrics & Gynecology Hospital of Fudan University. Informed consent from each patient has been obtained.

### Cell culture

A total of 7 human UCEC cell lines (RL95-2, HEC-1A, HEC-1B, Ishikawa, KLE, AN3-CA and ECC-1) were purchased from The Cell Bank of Type Culture Collection of The Chinese Academy of Sciences and their identity was confirmed by Shanghai Biowing Applied Biotechnology Co., Ltd. (Shanghai, China) via STR profiling. All cells were cultured in a humidified incubator at 37 °C with 5% CO_2_. Cell lines were cultured in RPMI-1640 medium (Gibco; Thermo Fisher Scientific, Inc., Carlsbad, CA, USA) supplemented with 10% FBS (Gibco; Thermo Fisher Scientific, Inc.), 100 units/ml penicillin and 100 μg/ml streptomycin (Gibco; Thermo Fisher Scientific, Inc.).

For recombinant lentiviral particle construction (GeneBio Co., Shanghai, China), FBXO17 gene expression plasmid was first constructed together with an empty plasmid (negative control). 293T cells were used to produce Ubi-FBXO17-3FLAG-CBh-gcGFP-IRES-puromycin lentiviral vector (LV-FBXO17) or Ubi-3FLAG-CBh-gcGFP-IRES-puromycin lentiviral vector (LV-NC) by collecting and filtering supernatants after 48 h of transfection. The FBXO17 stably overexpressing cell line (Ishikawa-oeFBXO17) was established by transfecting with LV-FBXO17; as a negative control, Ishikawa cells were transfected with LV-NC, and termed Ishikawa-NC. In detail, 30% confluent Ishikawa cells were incubated with LV-FBXO17 or LV-NC (MOI = 20) for 12h before changing the medium. After 72h of transfection, puromycin (2.5 μg/mL) was included to select stably transfected cells, which were identified using Western blot analysis and RT-qPCR analysis.

### Reverse transcription-quantitative (RT-q) PCR

Total RNA from cells was extracted using a RNeasy MiniKit (Qiagen, GmbH, Hilden, Germany) according to the manufacturer's protocol. Total RNA was reverse transcribed using PrimeScript™ RT MasterMix (Takara Bio, Inc., Shiga, Japan), followed by qPCR using Applied Biosystems™ QuantStudio™ 6 Flex (Thermo Fisher Scientific Inc., Waltham, MA, USA). The data were normalized to the housekeeping gene *GAPDH or ACTB*, and analyzed using the 2^-ΔΔCq^ method. The sequences of the primers were: *FBXO17* forward, 5'-TGGGGAAGATTGGGAAAGGC-3' and reverse, 5'-TCGCCCATTGGCTACATCTC-3' and *BAX* forward, 5'-CCCGAGAGGTCTTTTTCCGAG-3' and reverse, 5'-CCAGCCCATGATGGTTCTGAT-3' and *BCL-2* forward, 5'-GGTGGGGTCATGTGTGTGG-3' and reverse, 5'-CGGTTCAGGTACTCAGTCATCC-3' and *CASP3* forward, 5'-GAAATTGTGGAATTGATGCGTGA-3' and reverse, 5'-CTACAACGATCCCCTCTGAAAAA-3' and *GAPDH* forward, 5'-GTCAAGGCTGAGAACGGGAA-3' and reverse, 5'-AAATGAGCCCCAGCCTTCTC-3' and *ACTB* forward, 5'- GCCGACAGGATGCAGAAGGAGATCA-3' and reverse, 5'-AAGCATTTGCGGTGGACGATGGA-3' and *AXIN2* forward, 5'-CAACACCAGGCGGAACGAA-3' and reverse, 5'-GCCCAATAAGGAGTGTAAGGACT-3'.

### Western blot analysis

Cells and tissues were lysed on ice in RIPA buffer with 1:100 PMSF and 1:10 NaF for 30 min, and were then centrifuged at 14,000 × g for 20 min. A BCA protein assay was used to measure the concentration of the proteins. Protein samples were separated on an SDS-gel using SDS-PAGE (Beyotime Institute of Biotechnology, Shanghai, China), and transferred to a PVDF membrane. Subsequently, the membrane was blocked using 5% skimmed-milk/Tris-buffered saline Tween-20 (TBST) for 1 h, and incubated with the one of the following primary antibodies: Anti-FBXO17 (1:1,000; cat. no. 12844-1-AP; ProteinTech Group, Inc., Rosemont, IL, USA), anti-E-cadherin (1:1,000; cat. no. 3195; Cell Signaling Technology, Inc., Boston, MA, USA), anti-N-cadherin (1:1,000; cat. no. 13116; Cell Signaling Technology, Inc.), anti-β-catenin (1:1,000; cat. no. 8480; Cell Signaling Technology, Inc.), anti-cyclin D1 (1:20,000; cat. no. ab134175; Abcam, Cambridge, UK), anti-c-Myc (1:1,000; cat. no. 5605; Cell Signaling Technology, Inc.), anti-GSK3β (1:10,000; cat. no. ab32391; Abcam), anti-GSK3β (phospho S9) (1:10,000; cat. no. ab75814; Abcam), and anti-GAPDH (1:10,000; cat. no. 10494-1-AP; ProteinTech Group, Inc.), all of which were diluted in TBST. The membranes were next incubated with an HRP-conjugated secondary antibody (Beyotime Institute of Biotechnology), after which, signals were visualized using a UCECL Plus kit (Amersham, Buckinghamshire, UK). At least 3 independent Western blot experiments were performed.

### Cell Counting Kit 8 (CCK-8) assay

A CCK-8 assay (Shanghai Yeasen Biotechnology Co., Ltd., Shanghai, China) was used to determine cell growth. A total of 2.5x10^3^ cells (Ishikawa-oeFBXO17 or Ishikawa-NC) per well were plated into 96-well plates. Cell numbers were assessed in 24 h intervals. To measure cell proliferation, after incubation, 10 μl CCK-8 was added to each well to be measured and cultured for a further 2 h. Subsequently, the absorbance at 450 nm was measured using Synergy2 (BioTek China, Shanghai, China).

### Real-Time Cellular Analysis (RTCA)

RTCA was used to draw continuous proliferation curves. A total of 3x10^3^ cells were plated in 96-well microtiter E-plates and monitored using the xCELLigence RTCA DP instrument. The median and standard deviation were calculated from 3 individual replicate wells.

### EdU assay

A total of 1x10^6^ cells were plated in 6-well plates and cultured to ~50% confluence. Cells were then treated with 10 µM EdU (Invitrogen; Thermo Fisher Scientific, Inc., Carlsbad, CA, USA) for 30 min. An EdU analysis kit (Invitrogen; Thermo Fisher Scientific, Inc.) was used to detect EdU staining according to the manufacturer's protocol.

### Colony formation assay

A total of 800 cells were seeded in 6-well plates to form colonies. After 10 days of incubation, cells were fixed with 4% formaldehyde for 15 min and stained with 2% crystal violet for 20 min. The number of colonies in each group were counted using ImageJ (National Institutes of Health, Bethesda, MD, USA).

### Cell apoptosis and cell cycle analysis

Ishikawa cells were seeded into 6-well plates and cultured to 90% confluence. For cell apoptosis analysis, cells were stained using a PE Annexin V Apoptosis Detection kit I (BD Biosciences, Franklin Lakes, NJ, USA) according to the manufacturer's protocol. Then, flow cytometry analysis was performed to measure Annexin V-PE and 7-AAD-PC5.5 fluorescence values to identify early apoptotic cells. For cell-cycle analysis, cells were fixed using 70% ethanol overnight at 4 °C. Fixed cells were treated with 1 mg/ml RNase for 30 min at room temperature. Then PI (BD Biosciences) was used to stain the cells at 4 °C in the dark (30 min), followed by flow cytometry in the PE channel.

### Subcutaneous tumor implantation

Four-week-old female immune-deficient BALB/c-nu mice (weight, ~14 g) obtained from Animal Laboratory (Shanghai, China). Mice were housed at 22-24°C with an average humidity of 50-70%, a 12-h light/dark cycle and free access to food and water in a cage of 5 mice. For subcutaneous inoculation, 2x10^6^ Ishikawa-NC or Ishikawa-oeFBXO17 cells were injected into 10 mice (n = 5 per group; 2 groups). Animal health as well as tumor growth was monitored and measured every 3 days, and tumor volume was calculated using the following equation: Volume = 0.5 × (length × width^2^). Body weight loss > 20% or tumor diameter > 1.5 cm was assumed to be a humane endpoint for euthanasia. The percentage range of body weight increase was 28.1-48.9% within 27 days. All the mice were sacrificed under deep anesthesia (pentobarbital sodium, 50 mg/kg, intraperitoneal injection) by cervical dislocation after 27 days with the stop of both heartbeat and breathing taken as criteria for death, and tumors were extracted and weighed. All animal studies were approved by Ethics Committee for Animal Experimentation of Obstetrics & Gynecology Hospital of Fudan University and were performed in accordance with the guidelines described in the *Interdisciplinary Principles and Guidelines for the Use of Animals in Research, Testing, and Education*.

### Hematoxylin and eosin (H&E) staining and immunohistochemistry (IHC) analysis

Tumor tissues from mice were embedded in paraffin after fixing at 4 °C for 12 h. The blocks were cut into 4-μm-thick sections, which were then deparaffinized and dehydrated in a graded series of ethanol solutions. For H&E staining, these slides were observed under a microscope after staining. For IHC, they were first heated at 120 °C for 5 min for antigen retrieval, and soaked in 3% hydrogen peroxide for 20 min to quench endogenous peroxidase activity. After incubation with primary antibodies against FBXO17 (both for human and murine samples; 1:50; cat. no. 12844-1-AP; ProteinTech Group, Inc.) or Ki67 (for murine samples; 1:1,000; cat. no. ab15580; Abcam) or IgG control Polyclonal antibody (for human samples; 1:50; cat. no. 30000-0-AP; ProteinTech Group, Inc.) at 4 °C overnight, these slides were incubated with peroxidase labelled antibodies for 1 h. Finally, the color was developed using DAB and imaged under a microscope. IHC of the mice tissues was quantified using Image-Pro Plus software. The tissues were quantitatively scored to obtain the immunoreactive scores (IRSs) as described previously 28. Briefly, the IRS was the product of staining intensity, which was scored as 0, negative; 1, weak; 2, moderate; and 3, strong, and the percentage of immunoreactive cells, scored as 0, 0%; 1, 1%-25%; 2, 26%-50%; 3, 51%-75%; and 4, >75%. The IRS scores ranged from 0 to 12. The IHCs which scored ≥6 were considered as FBXO17-low expression and those with a score >6 were considered as FBXO17-high expression.

### TUNEL assays

Using a One-Step TUNEL Apoptosis Assay Kit (Beyotime Institute of Biotechnology), the apoptosis rate was detected in tissue slices. Briefly, slices were deparaffinised and rehydrated. After treatment with proteinase K (20 μg/ml) for 10 min at 37 °C, the slices were washed in PBS, and the labelling reaction was performed according to the manufacturer's instructions. The nuclei were counterstained with a DAPI staining solution (Beyotime Institute of Biotechnology) for 5 min at room temperature. The FITC-labeled TUNEL-positive cells were imaged by using 488-nm excitation and 530-nm emission.

### Protein-protein interaction (PPI) network construction and pathway enrichment analysis

PPI network, which included both genetic and physical interactions to FBXO17, were constructed using the Biological General Repository for Interaction Datasets database (BioGRID, version 3.5.173; www.thebiogrid.org). Then all the proteins in the results were performed a pathway enrichment analysis based on Kyoto Encyclopedia of Genes and Genomes (KEGG, http://www.genome.jp/kegg/) database using the Database for Annotation, Visualization and Integrated Discovery (DAVID) online tool (http://david.abcc.ncifcrf.gov).

### Statistical analysis

Statistical analysis was performed using SPSS version 22.0 (IBM Corp., Chicago, IL, USA). Quantitative data are presented as the mean ± standard deviation. Unpaired Student's t-test was used for the comparison of parameters between two groups. One-way ANOVA was used to compare the parameters among >2 groups. Association analyses of clinical factors were performed by Student's t-test (for measurement data such as age and BMI) or Pearson's χ^2^ test or Fisher exact-probability test (for enumeration data such as histology grade) using SPSS version 15.0 (SPSS, Chicago, IL, USA). Correlation analyses were conducted using the Pearson or Spearman method. P<0.05 was considered to indicate a statistically significant difference.

## Results

### FBXO17 expression is downregulated in UCEC

**Figure 1:**
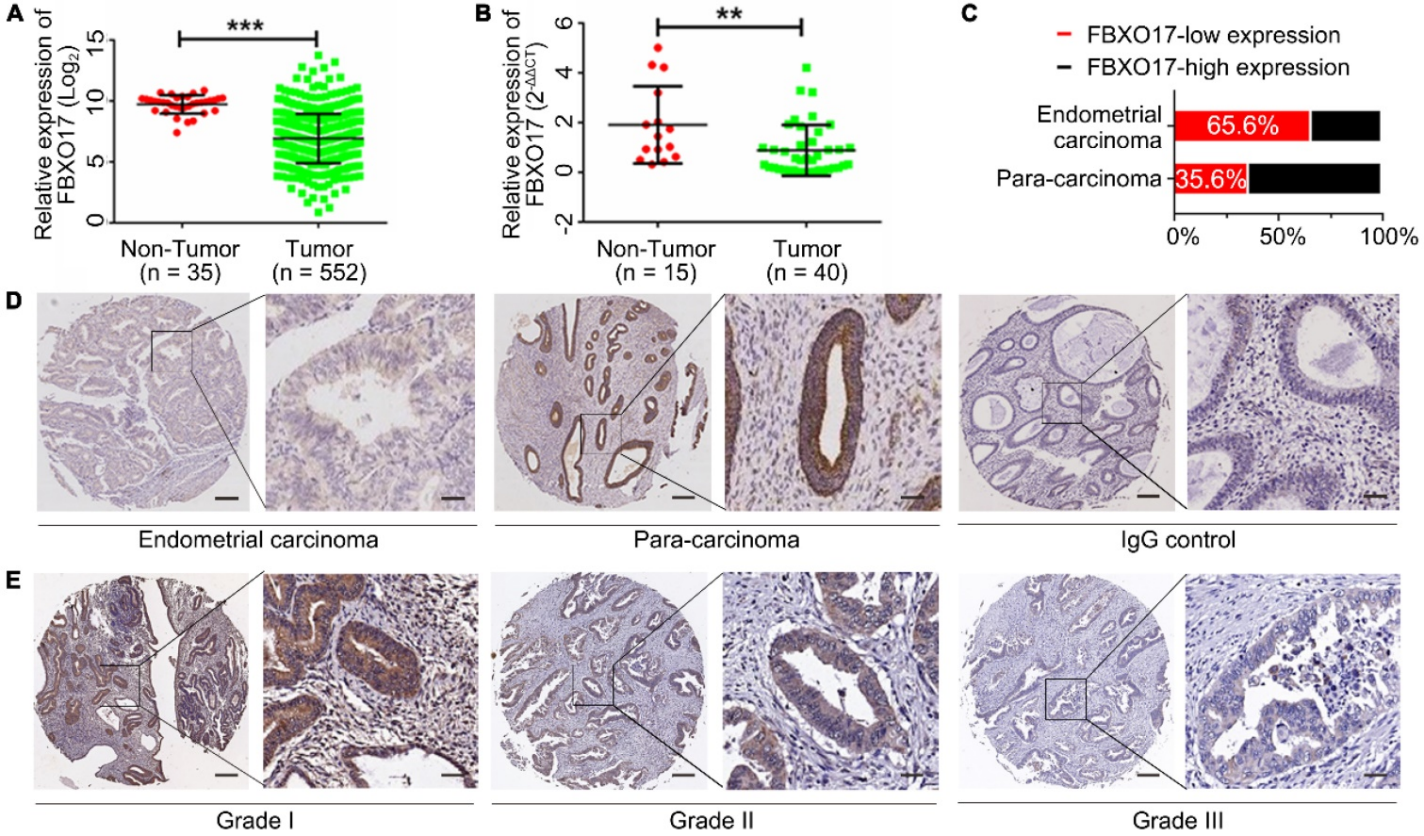
** FBXO17 had a low expression in the endometrial tumor.** Low FBXO17 mRNA expression in endometrial cancer from TCGA datasets **(A)** and Fudan cohort validation **(B)**. **(C-D)** Statistical data and representative IHC of FBXO17 expression in endometrial carcinoma and para-carcinoma. **(E)** Representative IHC of FBXO17 expression in different histology grades of endometrial carcinoma. Magnification, ×400 and ×60; scale bar: 200 µm and 50 µm. A high expression level of FBXO17 appears as brown particles. Data are mean ± SD, * P< 0.05, ** P< 0.01, *** P< 0.001. IHC: immunohistochemistry.

Using TCGA, the mRNA expression of FBXO17 in patients with UCEC was determined. The results of the analysis showed that FBXO17 mRNA was significantly lower in UCEC samples compared with the non-tumor endometrial tissues (P<0.001; Fig. 1A). Similar results were obtained from the cohort of patients recruited at Fudan University based on the RT-qPCR analysis (Fig. 1B). Additionally, markedly low expression of FBXO17 was observed in endometrial carcinoma tissues (n=90) using IHC compared with the paracancerous tissues (n=90; Fig. 1D). The proportion of FBXO17-low expression in endometrial carcinoma samples was significantly higher compared with the paracancerous tissue (65.6% vs 35.6%; Fig. 1C).

Clinical information (Table 2) confirmed the potential effect of downregulated FBXO17 expression on UCEC progression. Patients with a lower expression of FBXO17 had a higher histological grade (P<0.05, Fig. 1E). However, no difference was found in patients' survival between different FBXO17 expression groups based on the TCGA database, which might result from low sample size (Fig. S1).

### FBXO17 inhibits proliferation of Ishikawa cells

**Figure 2:**
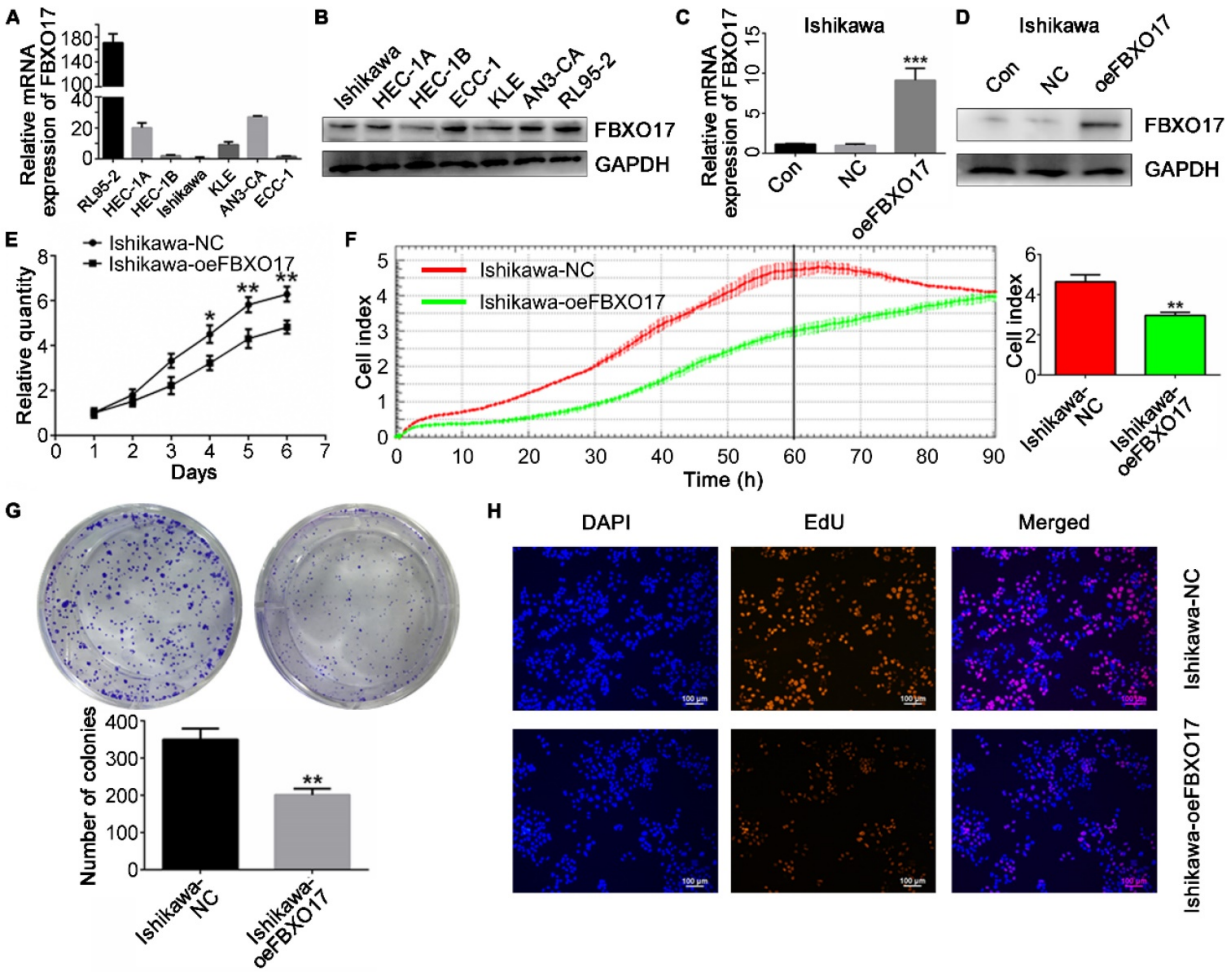
** FBXO17 inhibits proliferation of Ishikawa cells.** FBXO17 mRNA expression **(A)** and protein expression **(B)** in UCEC cell lines were detected. Ishikawa was selected for establishing cell line overexpressing FBXO17 (oeFBXO17) and **(C-D)** shows the overexpression efficiency, respectively by Western blotting and RT-qPCR. **(E-H)** Cell proliferation of Ishikawa after transfection of FBXO17 was shown by CCK8 assay (E), RTCA (F), colony formation assay (G), and EdU staining (H). Scale bar = 200 µm. All experiments were repeated at least three times. Data are mean ± SD, * P< 0.05, ** P< 0.01, *** P< 0.001. IHC: immunohistochemistry; UCEC: uterine corpus endometrial carcinoma; CCK8: Cell Counting Kit 8; RTCA: real-time cellular analysis.

Next, both the mRNA and protein levels of FBXO17 were measured in 7 UCEC cell lines (Fig. 2A and B). Ishikawa cells exhibited the lowest expression of FBXO17 amongst the cell lines and were thus used for all subsequent experiments. To determine the association between abnormal expression of FBXO17 and Ishikawa *in vitro*, an overexpression model (oeFBXO17) of the Ishikawa cell line was established using lentiviral transfection, and the transfection efficiency was detected (Fig. 2C and D).

**Figure 3:**
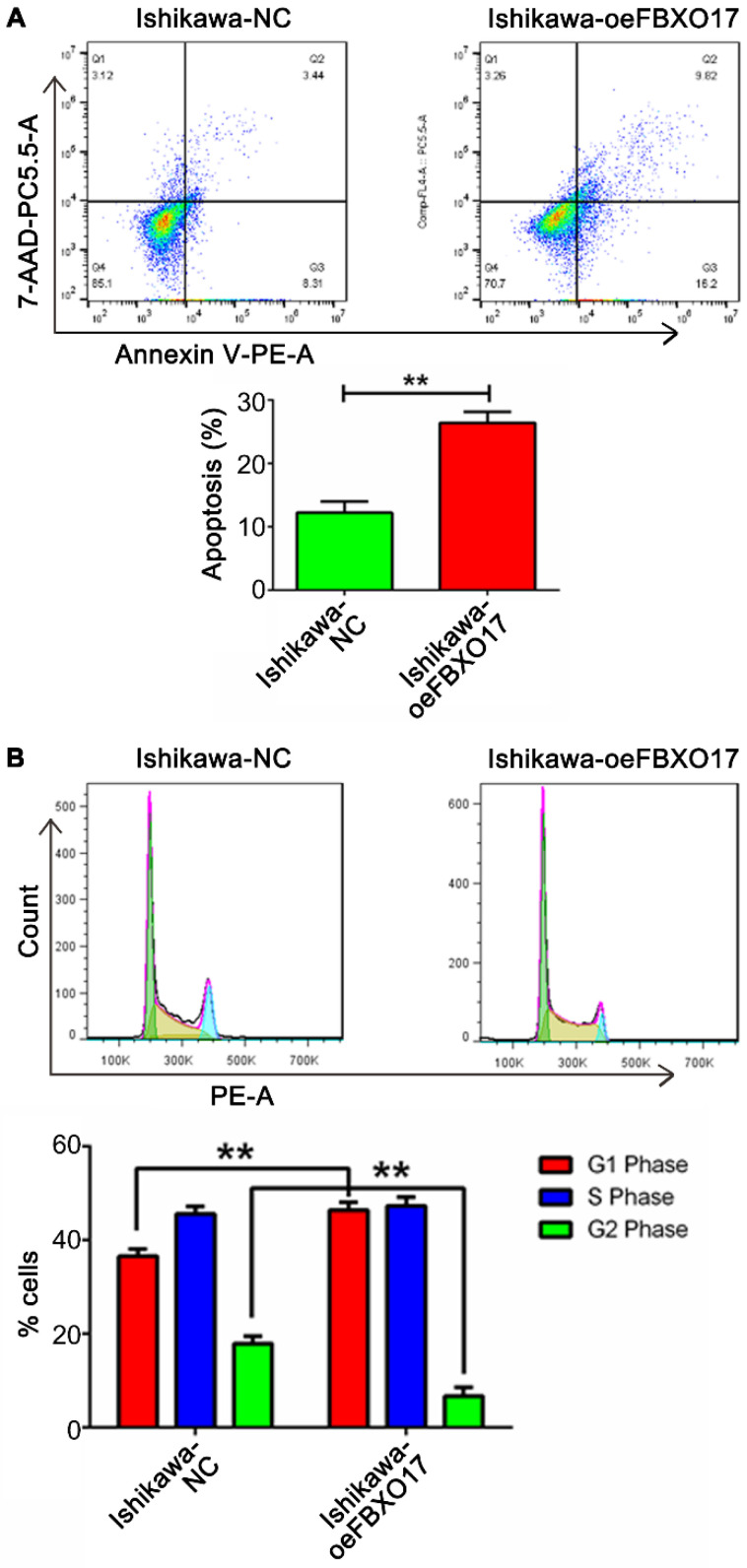
** FBXO17 promotes cell apoptosis and restrains cell cycle. (A)** Cell apoptosis of Ishikawa cells with overexpressed FBXO17 (Ishikawa-oeFBXO17) was much more activated compared with normal control (Ishikawa-NC). **(B)** Cell cycle distribution was detected by flow cytometry between the two groups. All experiments were repeated at least three times. Data are mean ± SD, * P< 0.05, ** P< 0.01, *** P< 0.001.

Cell proliferation was lower in the Ishikawa-oeFBXO17 cells compared with the Ishikawa-NC cells based on the CCK-8 assay after 4 days of culture (Fig. 2E). A similar trend of reduced growth of cells was observed using the RTCA assay, which showed the widest gap at ~60 h after inoculation (P<0.01; Fig. 2F). Additionally, UCEC cells overexpressing FBXO17 exhibited reduced growth based on the EdU staining and colony formation assays (Fig. 2G and H). Apoptosis was increased based on the results of the flow cytometry analysis (Fig. 3). Cell cycle analysis showed a significantly higher percentage of cells that were overexpressing FBXO17 in the G1 phase, and a lower proportion of cells in the G2 phase, suggesting that overexpression of FBXO17 disrupted the cell cycle. However, a change in the proportion of cells in the S phase was not observed.

Collectively, these results demonstrate that FBXO17 overexpression reduces proliferation. Therefore, downregulation of FBXO17 may promote proliferation and viability of Ishikawa cells *in vitro*.

### FBXO17 leads to the dysregulation of the Wnt/β-catenin pathway in Ishikawa cells

**Figure 4:**
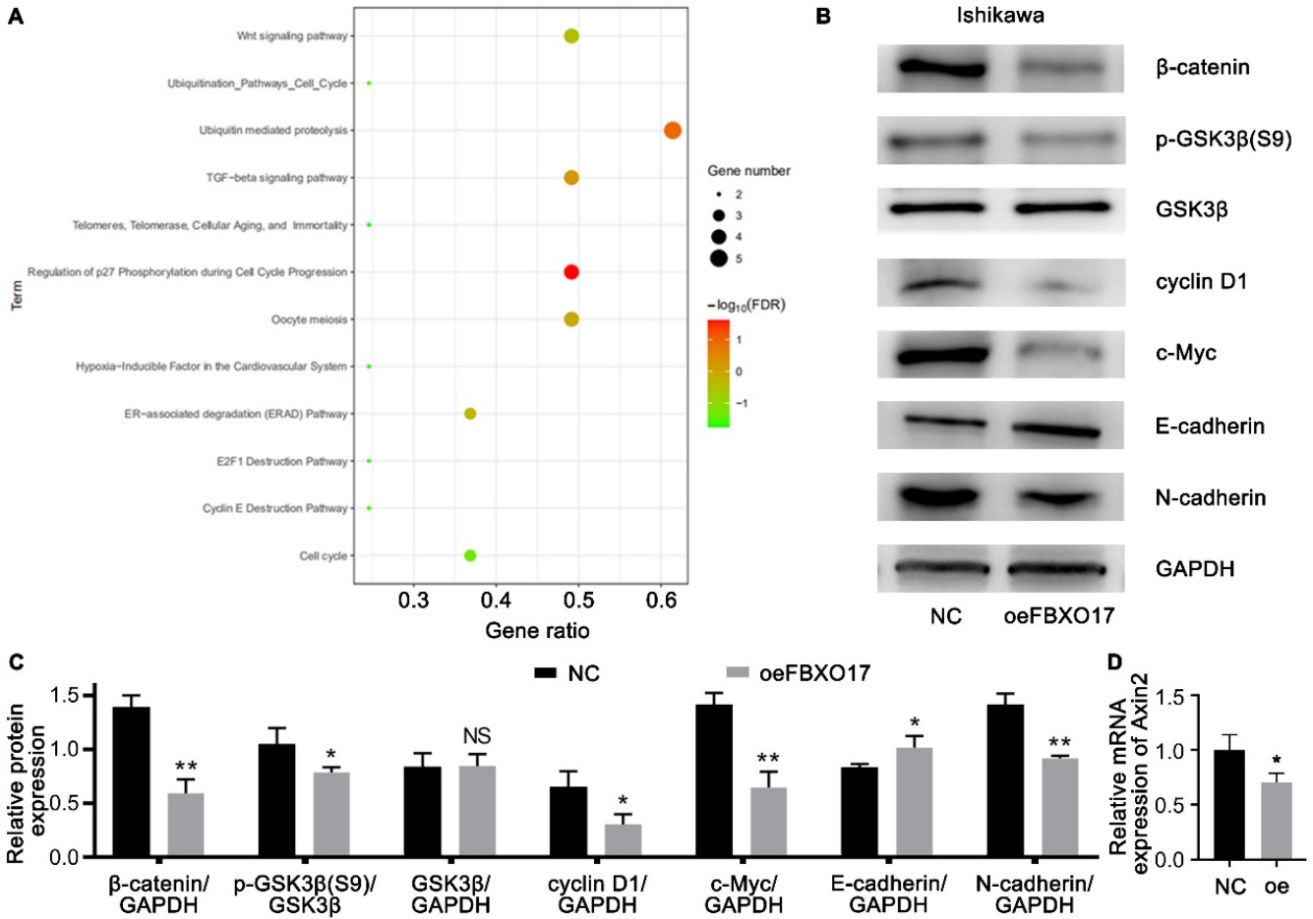
** FBXO17 leads to the dysregulation of the Wnt/β-catenin pathway in Ishikawa cells. (A)** Functional enrichment analysis of FBXO17 based on the GO database. **(B-C)** Ishikawa cells transfected with FBXO17 were tested for expression of β-catenin, GSK3β, p-GSK3β (S9), c-Myc, cyclin D1, E-cadherin and N-cadherin by western blotting. **(D)** Axin2 mRNA expression in Ishikawa cells transfected with FBXO17 were detected. Data are mean ± SD, * P< 0.05, ** P< 0.01, *** P< 0.001.

To explore the target genes that were possibly regulated by FBXO17, we used BioGRID for PPI analysis (Fig. S2) and DAVID for functional enrichment analysis based on the KEGG database. As shown in Fig. 4A, the KEGG analysis of FBXO17 were primarily enriched in the 'Wnt signaling pathway', 'ubiquitin mediated proteolysis', 'transforming growth factor-β (TGF-β) signaling pathway', 'regulation of p27 phosphorylation during cell cycle progression' and 'oocyte meiosis'. Since the Wnt signaling pathway has been found to be dysregulated in patients with UCEC 10, and previous studies have also reported the influence of FBXO17 on the Wnt pathway 24, 26, the Wnt/β-catenin was selected as a possible target pathway for subsequent experiments. The protein expression levels of β-catenin, GSK3β, pGSK3β, cyclin D1, c-Myc and the mRNA expression level of Axin2 were determined (Fig. 4B-D). Western blotting showed a lower level of β-catenin/GAPDH, GSK3β/pGSK3β, cyclin D1/GAPDH and c-Myc/GAPDH in the Ishikawa cells overexpressing FBXO17, and qRT-PCR showed a parallelly reduce in this group, suggesting that the Wnt signaling pathway was inhibited in these cells. Furthermore, an increase in E-cadherin expression and a decrease in N-cadherin expression were observed following overexpression of FBXO17. In order to verify if the interaction between β-catenin and E-cadherin caused the result 29, we used a co-staining assay and found that the two didn't show obvious difference in distribution between Ishikawa-NC and Ishikawa-oeFBXO17 cells (Fig. S3). These results indicated FBXO17 could disturb the Wnt/β-catenin pathway in UCEC.

### FBXO17 inhibits Ishikawa carcinogenicity *in vivo*

**Figure 5:**
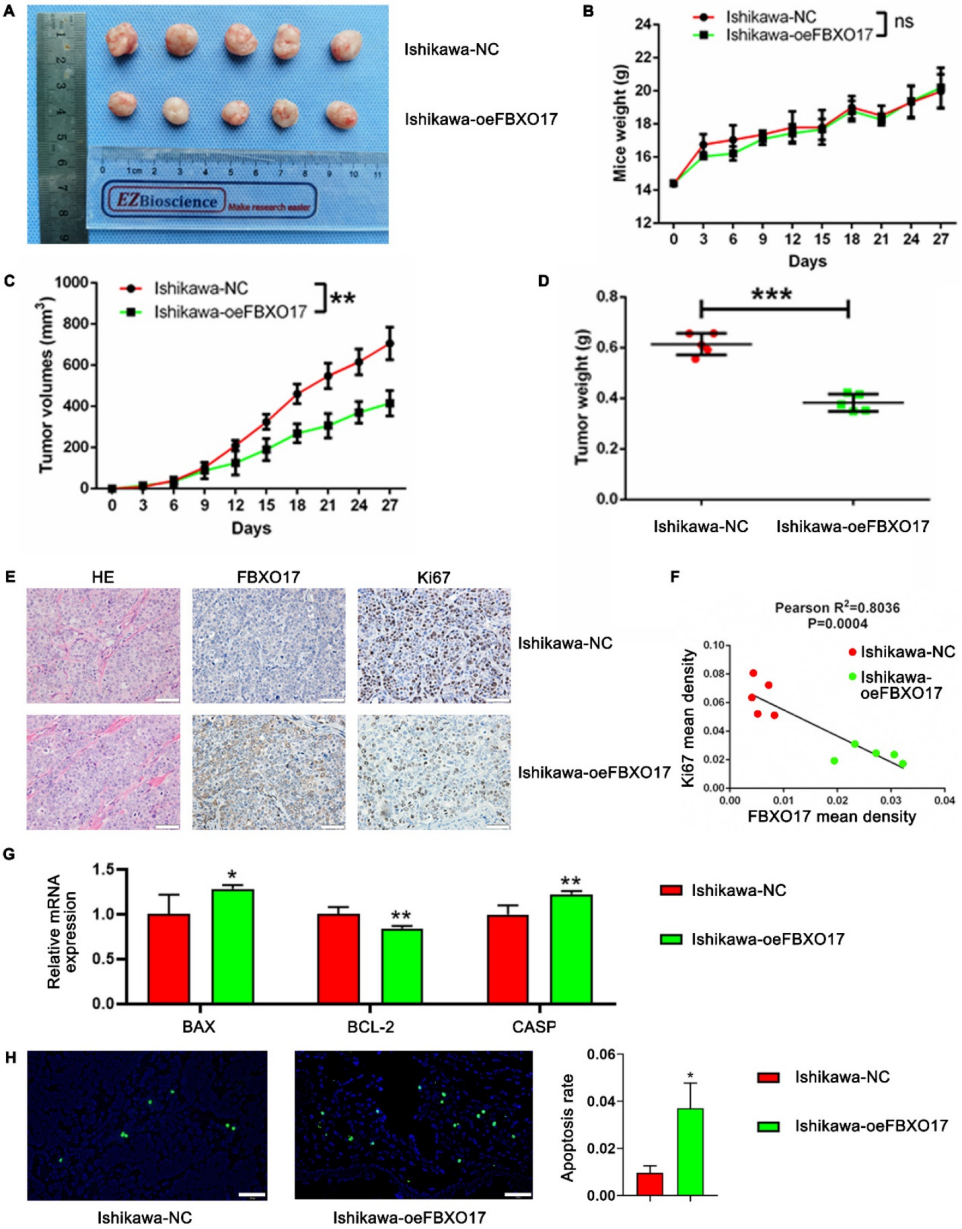
** FBXO17 inhibits Ishikawa carcinogenicity* in vivo*. (A)** Tumorigenicity assay in immune-deficient BALB/c-nu mice injected with Ishikawa cells. Mice weight **(B)** and tumor volumes **(C)** growth curves were drawn in 27 days. Final tumor weight was recorded on Day 35 (D) and the correlation analysis between the expression of FBXO17 and Ki67 was determined by IHC** (E-F)**. **(G)** The mRNA expression of BAX, BCL-2 and CASP3 in tumor tissues of the tumor-bearing mice. **(H)** Representative images show apoptotic cells in the tissue slices of the different groups of mice by TUNEL staining. Scale bar = 50 µm. Data are mean (n = 5) ± SD, * P< 0.05, ** P< 0.01, *** P< 0.001.

To further demonstrate the role of FBXO17 in tumor progression, a subcutaneous tumor implantation model using female nude mice was established. The tumor volumes of mice implanted with the FBXO17 overexpressing cells were significantly smaller than the mice implanted with the control cells (P<0.01), although the difference in the weight of the mice between the two groups was not significantly different (Fig. 5A-C). Tumor weight at the end of the experiment (day 27) was consistently lower in the FBXO17 overexpression group (P<0.001; Fig. 5D). The extracted tumor tissues were then sectioned for H&E staining and IHC analysis. The FBXO17 overexpressing group had a relatively low cancer cell density as well as Ki67 expression compared with the negative control group. Additionally, the mean density of Ki67 expression was negatively associated with that of FBXO17 expression, which indicated that increased FBXO17 expression was a negative factor for tumor proliferation (Fig. 5E-F). We have also tested the mRNA expression of apoptosis-relevant genes (BAX, BCL-2 and CASP3) in tumor tissues of our model mice (Fig. 5G), and a TUNEL assay in tissue slices (Fig. 5H), which revealed an elevated apoptosis tendency in FBXO17-overexpressed mice implanted tumor. Thus, FBXO17 upregulation in Ishikawa was confirmed to restrain carcinogenicity *in vivo*.

## Discussion

Several F-box proteins have been reported to influence the progress of cancers, of which only a few have been linked with UCEC. For example, FBXW7 was found to be mutated in ~16% of patients with UCEC, and this was associated with an increased rate of lymphatic metastasis 30, 31 In the present study, another F-box protein, FBXO17, was identified as a potential inhibitor of UCEC carcinogenicity. Decreased FBXO17 was observed at both the mRNA and protein levels in UCEC tissues and was confirmed to be associated with histological grade. According to previous studies, the dysregulation of F-box proteins in tumors may be ascribed to several causes, such as aberrant DNA methylation 32, 33, translational regulation by noncoding RNAs 34, gene mutation or CCAAT/enhancer-binding protein δ (CEBPδ) regulation under hypoxic conditions 35, but the regulation of FBXO17 in UCEC has not been previously shown, to the best of our knowledge.

Here, it was shown that FBXO17 reduced Ishikawa cell proliferation. Few studies have revealed the function of FBXO17 in cancer. Patients with elevated FBXO17 expression had a significantly shorter overall survival in high-grade glioma and esophageal squamous cell carcinoma 23, 32. Tomeka *et al* 27 recently evaluated FBXO17 expression in lung adenocarcinoma cells, where it was observed that it accelerated the progression of the tumor by regulating the PI3K-Akt-mTOR pathway.

It has been demonstrated that some F-box proteins function partly by affecting the Wnt signaling pathway, which is commonly activated in several types of cancer. In UCEC, Some F-box proteins can target and degrade β-catenin 36, 37. For example, Debasish *et al* 38 and Mohsina *et al* 39 found that FBXO16 could directly bind with β-catenin and promote its ubiquitylation and degradation, rather than targeting the upstream of the Wnt pathway like GSK3β. FBXO17 expression was found to be upregulated in hepatocellular carcinoma, and it regulated β-catenin, GSK3β, cyclin D1 and c-Myc function, suggesting that the entire Wnt axis was regulated by FBXO17 24. In the present study, the relationship between FBXO17 and the Wnt pathway in Ishikawa cells was assessed. Overexpression of FBXO17 resulted in reduced expression of the core factor β-catenin, p-GSK3β and transcription products (cyclin D1, Axin2 and c-Myc). One of the products, cyclin D1, which regulates the G1/S transition during the cell cycle, and the other, c-Myc, which acts as an important proto-oncogene, have both been found to be highly expressed in endometrial carcinoma and are positively correlated with tumor stage and survival 40-42. The results of the present study suggested that overexpression of FBXO17 in Ishikawa cells inhibited Wnt/β-catenin signal transduction activity, thereby decreasing the expression of cyclin D1, Axin2 and c-Myc.

We noticed that existing studies, though limited, have largely shown upregulated expression of FBXO17 in tumors 23, 24, 27. However, we could learn from other F-box proteins which are attenuated in some cancers. For example, low expression of FBXO16 could activate the Wnt signaling by degrading β-catenin in glioblastoma 39. Due to lack of further exploration for the mechanism in our and other studies, we could only explain the difference by the diversity of functions of the F-box family of proteins 24, 26, 27, 36. Since there was only primary verification at the protein level in the present study, additional studies are required to determine whether other pathways in addition to the Wnt pathway are involved in FBXO17-mediated reduction of proliferation.

Bioinformatics analysis also identified TGF-β signaling and p27 as possible targets regulated by FBXO17, and thus, the activity of these pathways should be studied further. Both TGF-β and p27 are regarded as important regulators of cell proliferation and cell cycle progression 43, 44. Several studies have shown that certain F-box proteins, such as β-TrCP1 and FBXO32, can downregulate Smad4, which is an important mediator of TGF-β signaling, resulting in decreased cell proliferation 45, 46. Amongst the various F-box proteins, Skp2 is a well-established oncogene that directly induces the degradation of p27 47. Based on these previous studies and the present study, the F-box proteins may exhibit the potential to influence multiple crucial processes in cell growth through ubiquitin ligases, a mechanism which may be used by FBXO17 as well.

In addition, the expression of FBXO17 was also related to E-cadherin and N-cadherin expression. Both of these cadherins are adhesion molecules, but with different functions. The expression of E-cadherin is lower in UCEC cells compared with the non-tumor endometrium, which leads to increased tumor invasiveness 48. N-cadherin is generally considered as a promoter of tumor aggressiveness 49. Whether FBXO17 can influence cell motility in UCEC still requires further study.

The proportion of EdU^+^ cells reflects the percentage of cells in S phase. In the present study, EdU staining showed a difference in the proportion of cells in the S phase, although no difference was observed based on the flow cytometry analysis. This difference may be due to the different cell status between the two experiments, as EdU staining requires space between cells for easy counting, whereas cell cycle analysis requires a relatively higher cell density.

There remains still uncertainty regarding targeting FBXO17 in UCEC as a treatment option, as given the complexity of the Wnt pathway, there are only a few clinically relevant Wnt inhibitors 5, 50. In the present study, the therapeutic potential of FBXO17 was not determined, and this will form the basis of future studies. However, it is well-established that the Wnt/β-catenin pathway is crucial for tumorigenesis, and based on the results of the present study, FBXO17 is associated with Wnt-mediated progression.

One of the shortcomings in this article is that we have only performed the overexpression experiment in one cell line of UCEC. The results in other cell lines or using the knockdown methods may be different considering the heterogeneity of the tumor. Comparing the different results will help to get a fuller understanding of the function of FBXO17 in UCEC.

In conclusion, the results of the present study showed the potential role of FBXO17 in Ishikawa cell proliferation and modulation of the Wnt/β-catenin pathway. These results highlight a novel means of regulation of the Wnt/β-catenin pathway in endometrial malignant lesions.

## Supplementary Material

Supplementary figures.

## Figures and Tables

**Table 1:** The clinical information of UCEC tissues (n=40) and non-tumor endometrium tissues (n=15)

Variables	Total	UCEC	Non-Tumor	*p* value
Age (years)		56.38±9.39	57.36±10.07	0.760
BMI		24.95±4.7	26.07±5.34	0.427
**Menopause**				0.716
Yes	43	32	11	
No	12	8	4	

UCEC: uterine corpus endometrial carcinoma; BMI: body mass index.

**Table 2:** FBXO17 expression in UCEC patients

Variables	Total	FBXO17 low	FBXO17 high	*p* value
	N	N	N	
Age (years)		57.7±8.26	59.48±11.40	0.399
BMI		25.04±4.5	25.41±4.3	0.695
**Menopause**				0.455
yes	71	33	38	
no	19	7	12	
**Myometrial invasion**				0.579
≤1/2	20	8	12	
>1/2	68	32	36	
**LVSI (+)**				0.134
no	80	34	46	
yes	8	6	2	
**Histology grade**				**0.038**
I	27	7	19	
II	47	24	23	
III	16	9	7	
**Lymph node metastasis**				0.141
no	51	29	22	
yes	4	4	0	
**Clinical stage**				0.187
I+II	75	31	44	
III+IV	15	9	6	

UCEC: uterine corpus endometrial carcinoma; BMI: body mass index; LVSI: lymph-vascular space invasion.

## Abbreviations

UCEC: uterine corpus endometrial carcinoma
GSK-3: glycogen synthase kinase-3
SCF: SKP1-Cullin1-F-box
TCGA: the cancer genome atlas
RT-q: reverse transcription-quantitative
CCK-8: Cell Counting Kit 8
RTCA: Real-Time Cellular Analysis
H&E: hematoxylin and eosin
IHC: immunohistochemistry
PPI: protein-protein interaction
DAVID: Database for Annotation, Visualization and Integrated Discovery
BioGRID: Biological General Repository for Interaction Datasets
TGF-β: transforming growth factor-β
CEBPδ: CCAAT/enhancer-binding protein δ
BMI: body mass index
LVSI: lymph-vascular space invasion
