# Supplementary material for: FBXO17 Inhibits the Wnt/β-Catenin Pathway and Proliferation of Ishikawa Cells
Source: Int J Med Sci. 2022 Aug 15;19(9):1430–41. doi: 10.7150/ijms.60335 (PMC9413558; doi:10.7150/ijms.60335)
Supplement: Supplementary file 1 — Supplementary figures. [file ijmsv19p1430s1.pdf]

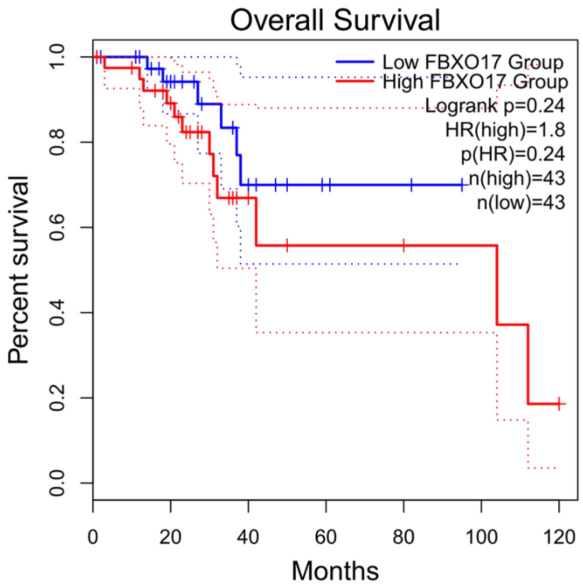

Figure S1. Kaplan-Meier survival curve of UCEC patients with high/low level of FBXO17 was drawn based on the TCGA database. UCEC: uterine corpus endometrial carcinoma; TCGA: The Cancer Genome Atlas.

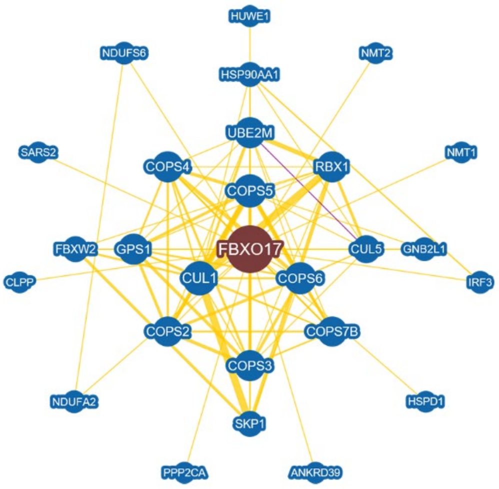

Figure S2. PPI network of FBXO17 downloaded from the BioGRID database. PPI: protein-protein interactions; BioGRID: Biological General Repository for Interaction Datasets.

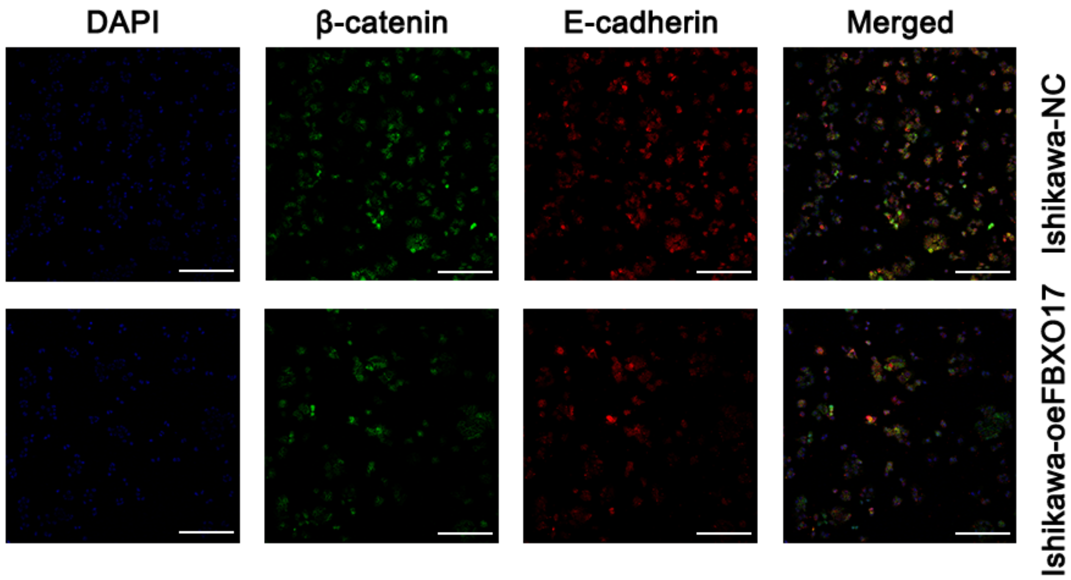

Figure S3. Ishikawa cells were immunostained with anti-β-catenin (red) and anti-E-cadherin (green) antibodies and visualized with confocal microscopy. DAPI (blue) was used to indicate cell nuclei. Scale bar = 250 μm.
